# Atypical Isolated Infections of the Infratemporal Fossa: A Diagnostic Challenge

**Published:** 2015-09

**Authors:** Sien Hui Tan, Aun Wee Chong, Narayanan Prepageran

**Affiliations:** 1 *Department of Otorhinolaryngology, Faculty of Medicine, University Malaya, Lembah Pantai, 50603 Kuala Lumpur, Malaysia*

**Keywords:** Aspergillosis, Infection, Maxillary sinus, Tuberculosis.

## Abstract

**Introduction:**

Atypical infratemporal fossa infections are rare and potentially fatal.

**Case Report:**

A case of an aspergillosis localized in the infratemporal fossa and another case of tuberculosis of the infratemporal fossa originating from the maxillary sinus, is described. The first patient was immunocompromised and showed symptoms of facial numbness; whereas the other was an immunocompetent man who complained of trigeminal neuralgia type pain. It was difficult to differentiate between infection and tumour despite the utilization of computed tomography scans and magnetic resonance imaging.

**Conclusion:**

These cases illustrate the need for a high index of suspicion; in addition to endoscopic confirmation and histopathology to establish precise diagnosis and early intervention.

## Introduction

Atypical infratemporal fossa (ITF) infections are extremely uncommon and can be potentially fatal. They have been known to arise from odontogenic infections, dental extractions, maxillary sinus wall fractures, and maxillary sinusitis (1-5). Two unique cases of isolated infections involving the ITF are described. The first is an aspergillosis localised to the ITF and the second is tuberculosis (TB) of the ITF originating from the maxillary sinus. To the best of our knowledge, no such cases have been reported in current literature.

## Case Reports

**Case 1:** A 37–year-old immunocompro- mised post renal transplant man presented himself to the Otolaryngology Department with a four-month history of left facial numbness and left upper molar pain that persisted despite dental extraction. There were no other otorhinolaryngological symptoms. Past medical history revealed that the patient was on an immuno- suppressive regime and suffered from hypertension. 

**Fig. 1:**
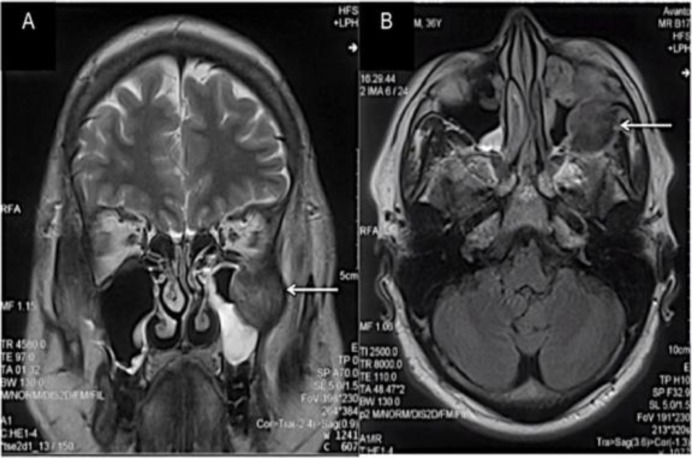
Coronal (A) and axial (B) MRI scans demonstrating a soft tissue lesion infiltrating the temporalis and lateral pterygoid muscle in the left infratemporal fossa

Clinical examination and flexible nasoendoscopy findings were unremarkable. Computed tomography (CT) scan and magnetic resonance imaging (MRI) reported an irregular soft tissue infiltrating the temporalis and lateral pterygoid muscle (Fig. 1). There was no intracranial extension.

The pre-operative diagnosis was a left maxillary sinus tumour. The patient underwent a left endoscopic excision of the tumour via maxillotomy. Peri-operatively, it was noted that the lesion had a light yellowish colour and was solely confined to the ITF with no involvement of the maxillary sinus. Histopathological examination reported granulation tissue with inflammatory cells and fungal hyphae. Apergillus fumigatus was confirmed during fungal culture of the specimen. The final diagnosis was localised aspergillosis of the ITF.

Post-operative recovery was uneventful and the patient was commenced on a six-week course of voriconazole. As there was minimal improvement in the patient’s condition and slight reduction in the size of the mass, he underwent further debulking via an extended endoscopic medial maxillectomy. He was followed up for a year with no signs of recurrence.

**Case 2**: A 56-year-old immunocompetent man presented himself to the Otolaryngology Department with a three-month history of left facial pain and swelling, limited mouth opening, and left upper molar pain, which persisted despite dental extraction. There were no other otorhinolaryngological symptoms. Past medical history was unremarkable with no apparent immune deficiencies.

**Fig 2:**
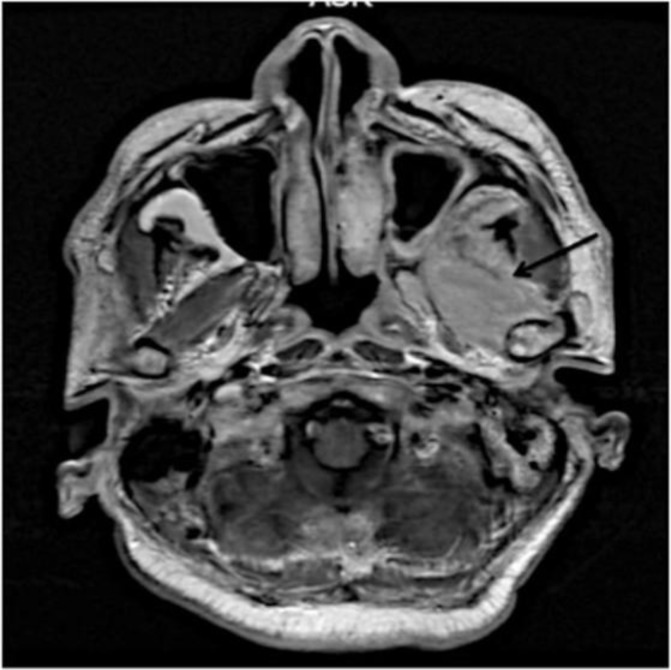
Axial MRI scan demonstrating extensive edema of the left lateral pterygoid muscle in the left infratemporal fossa.

Clinical examination revealed marked trismus with interincisal opening of 4 mm, making intra-oral examination difficult with an indurated swelling over his left maxillary region. Flexible nasoendoscopy showed medialisation of the left medial wall of the maxilla. CT scan and MRI reported a soft tissue mass in the maxillary sinus with involvement of the left pterygoid muscles and left masseter muscle (Fig.2). There was no intracranial extension. The pre-operative diagnosis was a left maxillary sinus tumour. The patient underwent a left endoscopic excision of the tumour via maxillotomy. Peri-operatively, it was noted that the lesion was located in the maxillary antrum with mild erosion of the posterior wall and a biopsy was performed. Histopathological and microbiological examination confirmed TB. Further investigations showed no evidence of systemic TB. The final diagnosis was TB of the ITF originating from the maxillary sinus.

Post-operative recovery was uneventful. The patient was started on an antitubercular regime and was followed up for a year with no recurrence.

## Discussion

The ITF is defined as a space bound anteriorly by the posterior surface of the maxilla, posteriorly by the mastoid and tympanic portions of the temporal bone, superiorly by the greater wing of the sphenoid and the squamous portion of the temporal bone, medially by the pterygoid process of the sphenoid, laterally by the zygomatic arch and ascending ramus of the mandible, and inferiorly by the posterior belly of the digastric muscle and the angle of the mandible. Its contents are the temporalis muscle, medial and lateral pterygoid muscles, pterygoid venous plexus, the maxillary artery and its branches, the mandibular nerve and the chorda tympani.

The presence of ITF infections is largely determined by which part of the anatomy is affected. Facial swelling can manifest as a result of an abscess occupying the ITF and fullness of this area is usually apparent on clinical examination. Trismus also occurs as a result of extension to the pterygoid muscles. Additionally, there may be trigeminal neuralgia type pain, hypothesia or paraesthesia that is indicative of the mandibular nerve involvement. Interestingly, both these patients presented different symptoms, with one complaining of facial numbness whereas the other had facial swelling and trigeminal neuralgia type pain. Hence, even if the clinical signs and symptoms are rational from an anatomic basis, the diagnosis of ITF infections remains a challenging task as they can present themselves in a variety of ways.

Imaging studies are fundamental for diagnosis and surgical planning. CT can distinctly distinguish between cellulitis and abscess formation whereas MRI is better for assessing soft-tissue. Nevertheless, it is often difficult to differentiate between infection and tumour, particularly in these cases where radiological and clinical features are nonspecific. Chronic infections may mimic neoplasm by showing signs of trismus and swelling without fever; as opposed to acute infections where there is usually pain and fever. In fact, our initial impression was a maxillary sinus tumour. As such, endoscopic confirmation with histopathology and culture are necessary for a definitive diagnosis. 

Achieving the correct diagnosis is crucial so that effective therapy can be tailored according to the pathology. In the first case of aspergillosis, surgical treatment was necessary when medical therapy alone failed. It is concluded that endoscopic debridement combined with systemic antifungals is imperative to improve survival, especially in immunocompromised patients. The second patient with TB improved significantly once he was commenced on an antitubercular therapy. 

To the best of our knowledge, the first case of aspergillosis solely localized in the ITF is reported. Goyal et al reported four patients presenting invasive fungal sinusitis with infratemporal fossa extension (6). The route of infection is via haematogenous spread or by direct extension from the sinuses, bones and external ear. Our patient had no evidence of systemic disease and this exceptionally atypical presentation can be attributed to his immunocompromised status. Weiland et al reported aspergillosis as a fatal disease in renal transplant patients (7), with a mortality rate of 68%. 

Our second patient presented an exceedingly rare case of TB in the ITF with the primary involvement of the maxillary sinus. Very few cases, documented in literature, show the maxillary sinus as the primary site of infection (8-10). The disease is almost always a consequence of pulmonary or extrapulmonary TB reaching the paranasal sinus via the haematogenous route or by direct extension. The onset of the human immunodeficiency virus (HIV) epidemic has caused an unexpected resurgence of TB and this is made worse by the emergence of multi drug resistant strains. It can be easily missed as a diagnosis in an atypical setting, particularly for our second case who was an immunocompetent man with no history of pulmonary TB. 

The venous drainage of the ITF is done through the pterygoid plexus, which anastomoses with the cavernous sinus, ophthalmic veins, and pharyngeal venous plexus. As a result, infections involving the ITF can spread haematogenously into the cavernous sinus and orbit leading to ominous consequences (11). Infections can also transverse to the adjacent fascial spaces leading to complications such as mediastinitis, pericarditis, and even death. Hence, it is imperative to avoid a delay in diagnosis and commence treatment promptly.

## Conclusion

These cases illustrate the need for a high index of suspicion to achieve a precise diagnosis so that the treatment can be tailored according to the pathology. We believe that prompt diagnosis of the disease based on radiological and pathological findings and early intervention are crucial in establishing a positive outcome.

## References

[1] Schwimmer A, Roth SE, Morrison SN (1988). The use of computerized tomography in the diagnosis and management of temporal and infratemporal space abscesses. Oral Surg Oral Med Oral Pathol.

[2] Leventhal D, Schwartz DN (2008). Infratemporal fossa abscess. Arch Otolaryngol Head Neck Surg.

[3] Gallagher J, Marley J (2003). Infratemporal and submasseteric infection following extraction of an infected maxillary third molar. Br Dental J.

[4] Weiss BR (1977). Infratemporal fossa abscess unusual complication of maxillary sinus fracture. Laryngoscope.

[5] Raghava N, Evans K, Basu S (2004). Infratemporal fossa abscess: complication of maxillary sinusitis. J Laryngol Otol.

[6] Goyal P, Leung M, Hwang PH (2009). Endoscopic approach to the infratemporal fossa for treatment of invasive fungal sinusitis. Am J Rhinol Allergy.

[7] Weiland D, Ferguson RM, Peterson PK, Snover DC, Simmons RL, Najarian JS (1983). Aspergillosis in 25 renal transplant patients. Ann Surg.

[8] Gleitsmann JW (1907). Tuberculosis of accessory sinuses of the nose. Laryngoscope.

[9] Shukla GK, Dayal D, Chabra DK (1972). Tuberculosis of maxillary sinus. J Laryngol Otol.

[10] Singh S, Singh A (1997). Primary tuberculosis of maxilla. Indian J Otolaryngol Head Neck Surg.

[11] Headley DB, Dolan KD (1991). Infratemporal fossa abscess. Ann Otol Rhinol Laryngol.

